# Sleep Medication and Athletic Performance—The Evidence for Practitioners and Future Research Directions

**DOI:** 10.3389/fphys.2016.00083

**Published:** 2016-03-07

**Authors:** Lee Taylor, Bryna C. R. Chrismas, Ben Dascombe, Karim Chamari, Peter M. Fowler

**Affiliations:** ^1^Qatar Orthopedic and Sports Medicine Hospital, Athlete Health and Performance Research Centre Aspire ZoneASPETAR, Doha, Qatar; ^2^Sport Science Program, College of Arts and Sciences, Qatar UniversityDoha, Qatar; ^3^Department of Rehabilitation, Nutrition and Sport, School of Allied Health, LaTrobe UniversityMelbourne, VIC, Australia

**Keywords:** sleeping pills, exercise, hypnotics and sedatives, medication, athletes, travel, practice, practitioner

Sleep is a restorative circadian process underpinned by numerous interrelated biological processes (Brown et al., 2012; Goel et al., 2013; Zeitzer, 2013; Vyazovskiy and Delogu, 2014). Specifically, biological rhythms in core temperature, blood pressure, immune function as well as melatonin, and other hormones demonstrate an intricate relationship with sleep (Zisapel, 2007). Therefore, whilst sufficient sleep [7–9 h is recommended by the National Sleep Foundation (NSF) for adults (Hirshkowitz et al., 2015)] facilitates an optimal internal temporal order, an increased risk of poor quality of life, morbidity, and mortality (Zisapel, 2007) are seen with insufficient sleep (<6 h is not recommended by the NSF for adults), which is currently a concern in both the general population and athletes (Halson, 2015).

Recent reviews (Fullagar et al., 2015a,b; Nédélec et al., 2015a; Thun et al., 2015) outline the proposed importance of sleep for athletes to reduce illness and injury rates (Luke et al., 2011; Milewski et al., 2014) whilst optimizing recovery and/or subsequent athletic performance (see Fullagar et al., 2015b for definition of terms). Moreover, the need for sufficient sleep to promote general mental and physical development within adolescent athletes has recently been highlighted by the International Olympic Committee (Bergeron et al., 2015). Practitioners and clinicians are thus under increasing pressure to encourage sufficient sleep in their athletes, since inadequate sleep has been reported to reduce various physical performance measures (Bulbulian et al., 1996; Souissi et al., 2003; Blumert et al., 2007; Oliver et al., 2009; Skein et al., 2013; Smith et al., 2013; Fullagar et al., 2015a; Thun et al., 2015). However, a dose response relationship between sleep loss and performance decrement has not been well established.

Limited evidence is available regarding the negative influence of circadian desynchrony (Smith et al., 2013), sleep disturbance (Souissi et al., 2013), and sleep deprivation (Skein et al., 2013) on athletic performance (see recent reviews Fullagar et al., 2015a,b). However, pharmacologically mediated improvements in sleep quality in the presence, or not, of these challenges is sparse within healthy athletic populations, as are data regarding any pharmacologically mediated athletic performance “hangover” effects within or next day (as discussed later in the present opinion article). Despite this limited evidence anecdotal reports from the media and those involved within professional sport suggest increased pressure on the medical practitioner to prescribe sleep medications. This pressure can be exerted from team managers, coaches and from the athletes themselves. Evidently there is likely disconnect between evidence and current practice.

Professional sportspeople (both players and officials) face unique challenges relative to their ability to achieve sufficient sleep (Sargent et al., 2014; Bergeron et al., 2015; Fullagar et al., 2015b; Juliff et al., 2015; Lastella et al., 2015a,b; Nédélec et al., 2015a; Thun et al., 2015). For example, inter- and intra-continental travel is common (McGuckin et al., 2014), with athletic performance often proximal to both departure and return travel (Reilly et al., 2005; Richmond et al., 2007; Pipe, 2011; Schwellnus et al., 2012). Alternating training and competition timings can be experienced by athletes, with morning, afternoon, and evening competition and training (Eagles et al., 2014; Meyer et al., 2014; Fullagar et al., 2016) often evident in a non-cyclical manner across a season (Fullagar et al., 2015b). On- and off-field competition demands can also impact sleep, especially during fixture congestion and ultra-competitive periods of the season (Murray et al., 2014; Carling et al., 2015; Dellal et al., 2015; Juliff et al., 2015), which are compounded by everyday life and family demands. Interestingly, individual athletes were shown to retire and wake earlier whilst obtaining less sleep (~ –0.5 h) than athletes from team sports (Lastella et al., 2015b). Whilst the night prior to (~ –0.9 h) and on the first evening of competition (~ –0.6 h) cyclists achieved less sleep than compared to pre-competition baseline values (~ 7.4 h; Lastella et al., 2015a). Such data (Lastella et al., 2015a,b) reinforces the need for individualized sleep maintenance/enhancement strategies within and between athletic disciplines (Fullagar and Bartlett, 2015) whilst indicating why sleep medications are utilized prior to and within competition phases. Some religions also face unique challenges, for example during Ramadan, training, nutritional, and prayer demands interact to negatively influence sleep (Roky et al., 2001; Reilly and Waterhouse, 2007; Zerguini et al., 2007; BaHammam et al., 2010; Chamari et al., 2016). Empirical data demonstrates Ramadan fasting reduces sleep duration [with (~ –1.13 h) and without napping (~ –1.29 h)] whilst delaying bedtime by ~1.32 h, when compared to non-fasting base-line control values (BaHammam et al., 2013). Indeed, Ramadan fasting was recently shown to double night awakenings (~2 vs. 4), increase light sleep (~2.5 h vs. ~1 h), reduce deep (~1.5 h vs. ~0.5 h), and rapid eye movement (~1.5 h vs. ~0.6 h) sleep, compared to baseline control values, respectively, within trained cyclists (Chamari et al., 2016). Therefore, achieving sufficient sleep for consecutive days is evidently challenging for athletes across the globe.

To address this challenge, three main intervention types have been explored/prescribed by sports medicine/science practitioners; pharmacological (Reilly et al., 2001), nutritional (Halson, 2008, 2014; Costello et al., 2014), and sleep hygiene (Fowler et al., 2015; Nédélec et al., 2015b). Pharmacological interventions are utilized despite the paucity of evidence regarding their efficacy relative to sleep within healthy populations (Paul et al., 2001, 2003, 2004a,b). In addition, much of the data has limited external validity to practice for a multitude of reasons, including the participant population (e.g., insomniacs, Buscemi et al., 2007; Vandermeer et al., 2007; Sullivan, 2012; Zisapel, 2012; Riemann et al., 2015; rather than healthy individuals and/or athletes) and outcome measures used (i.e., lack of familiarization, no placebo, and/or true control condition and lack of sport specific performance tests). Of further concern is recent anecdotal evidence suggesting that sleep medication overreliance/abuse is frequent in elite athletes, including off-prescription non-physician approved use and/or physician prescribed approved use. Therefore, this opinion article seeks to discuss specific future research directions related to the sleep medication that practitioners and athletes are currently utilizing, particularly how the current literature lacks external validity for sport medicine/science practitioners. It aims to suggest data which researchers could develop in the future to underpin their practice, relative to sleep medication use and subsequent within or next day athletic performance.

The incidence of prescribed and over-the-counter sleep medication use within professional sport remains to be reported upon, though data from collegiate athletes indicates sport-specific differences. A 2014 report from the National Collegiate Athletic Association (NCAA) indicated that male and female swimmers had the highest incidence of sleep medication use (18.2 and 16.9%, respectively) compared to any other sport assessed, with such medication accounting for 10.3% of miscellaneous substance use across all sports (Rexroat, 2014). Recreational sleep medication [n.b. sleep medications are not presently on the 2016 World Anti-Doping Agency (WADA) list] use in conjunction with energy drinks and/or alcohol to produce a chemical “high,” akin to the effects of some recreationally abused drugs (which are evidently WADA prohibited) has been reported within elite athletes, and is evidently of concern to practitioners. Indeed, Olympic champions have openly been placed into drug rehabilitation due to dependence on sleep medication, with a high profile Olympic nation recently banning all sleep medication use by their athletes once selected for Rio 2016; advancing their previous ban for certain hypnotic medications employed only 3 weeks prior to London 2012.

The concept of “appropriate” sleep medication use by athletes and practitioners is typically categorized under three circumstances; (i) pre/post competition or post training to reduce arousal (sedative action), (ii) to combat jet-lag (chronobiological purpose), and (iii) for habitually poor sleepers (manage insomnia). Sports medicine/science practitioners and their athletes have various classes of sleep medication to rely upon. Those drugs requiring a prescription and used on-label are ligand γ-aminobutyric acid (GABA) agonists or receptor modulators, benzodiazepine hypnotics (for simplicity z-drugs are included within this class), melatonin receptor agonists (principally Circardin®) and off-label use of sedative anti-depressants. Over-the-counter sleep medications are typically antihistamine based, with some off-label use of allergy specific drugs seen. Pharmacological review of each of these medications is beyond the scope of this opinion piece, instead, practitioners are directed to recent reviews for this information (Buscemi et al., 2007; Vandermeer et al., 2007; Sullivan, 2012; Zisapel, 2012; Costello et al., 2014).

Within healthy volunteers (i.e., those without clinically diagnosed insomnia) the next day (or upon awakening) effects of various sleep medications on psychomotor performance have been reported (Paul et al., 2003, 2004a,b). However, within athletic populations, intermittent high-intensity, prolonged endurance, and resistance based exercise, post ingestion of sleep medications, has not been extensively explored without the presence of confounding factors. Ecologically, the residual effects of such medication administered the evening before competition/training and their influence on subsequent athletic performance (i.e., the next day) is relatively unknown. For example, the efficacy of these medications to positively influence sleep duration, efficiency, latency, and inertia, together with perceived sleep quality, is limited within apparently healthy and/or athletic (particularly elite) populations. Instead data is often ascertained from clinically diagnosed sufferers of insomnia (Buscemi et al., 2007; Vandermeer et al., 2007; Sullivan, 2012; Zisapel, 2012; Riemann et al., 2015) and/or from non-athletic but apparently healthy populations (Paul et al., 2001, 2003, 2004a,b); thus a disconnect between evidence and practice is seen. Within (1.25 and 5.25 h post-ingestion; Atkinson et al., 2005) and next day (~9 h post-ingestion; Atkinson et al., 2001), negligible improvements were reported in physical performance (4 km cycling time trial and grip strength) following melatonin (5 mg) ingestion within physically active males. However, there were notable decrements in alertness, reaction time and short-term memory recall, despite no improvement in subjective sleep quality when melatonin was ingested 30 min before bed time. Other related experimental designs (efficacy of sleep medication upon sleep quality and any performance “hangover” effects) utilizing eight male University volleyball team players have been reported, though a lack of robust familiarization across the physical and cognitive tests employed and the non-elite status of the participants, preclude data inferences from secure use by practitioners (Ito et al., 2007). Lastly, similar examples of exercise performance post-sleep medication ingestion have been conducted at altitude, yet these do not utilize robust familiarized, reliable, and externally valid assessments of physical performance and evidently the confounding influence of sojourn to various altitude elevations is present (Beaumont et al., 2004; Nickol et al., 2006; Jouanin et al., 2009).

Simple placebo, double-blind experimental designs, with a true control condition, exploring sleep medication use and its influence on familiarized and reliable subsequent (or within) day athletic performance (i.e., influence of sleep medication on performance trials compared to pre-medication baseline; Fullagar et al., 2015b) are therefore required for practitioners to base their practice upon. Moreover, specific emphasis on athletic populations (ideally elite athletes) within appropriate age boundaries, at sea-level and without chronobiological disturbances is warranted. It is only once this basic evidence is compiled that more complex designs can be adopted. Evidently, there are a plethora of different sleep medications (see recent reviews Buscemi et al., 2007; Vandermeer et al., 2007; Sullivan, 2012; Zisapel, 2012; Costello et al., 2014), complicated by brand names and their variation within and between countries. Hence, practitioners should be directed by medically qualified staff with sufficient expertise relative to such medication. Researchers exploring this paradigm should also collaborate with medically qualified support staff, athletes and relevant practitioners, discussing the efficacy and external validity of medication options, with attention to potency, half-life and dosage key to maximizing external validity and reducing next (or within) day performance decrement risk. The use of such medication would evidently have to be flexible regarding the drug of choice, given the changing training and competition demands across a season (e.g., an English Premier League soccer season, with competing UEFA Champions League demands), chronotype variation (Waterhouse et al., 2002; Facer-Childs and Brandstaetter, 2015), and individual responses to different drugs and dosages (Evans and Johnson, 2001; Zhang and Dolan, 2010; Rissmann et al., 2012). Greater evidence for practitioners, enhanced interaction between medically qualified staff, athletes and practitioners, and tri-partite education within this axis, would hopefully reduce self-administration and off-label use of such medications by athletes, especially given the affinity for some of these medications to induce dependency in a matter of weeks and the reported “recreational” use of these drugs by some elite athletes.

Relative to this call for data, it is important that aside from residual performance decrements, the general and future health of athletes is of concern to practitioners. Some of these medications can induce dependency in a matter of weeks, and other health concerns within the clinical literature (though typically related to older members of society) are suggestive of an increased cancer risk (Kripke et al., 2012), accidental over-dose, and dangerous drug interactions particularly when opioid based analgesia is in use (Park et al., 2015). Lastly, some unexpected risks for athletes, and the general public, regarding sleep medication use are also present, e.g., new use of sedative hypnotics is associated with an increased risk of a motor vehicle crash (Hansen et al., 2015).

Therefore, to summarize the authors believe that the following research questions should be explored, in order to provide evidence for practitioners to (a) utilize within their practice and (b) stimulate further more complex research designs:

- Current sleep medication (and other sleep interventions) use within elite sport, similar to the NCAA data discussed (Rexroat, 2014).
- Sleep medication use within athletic populations (ideally elite athletes) and their impact upon sleep duration, efficiency, latency and inertia together with perceived sleep quality, with efficacy assessed by polysomnography within a laboratory environment and actigraphy within applied settings (Leeder et al., 2012).
- Sleep medication use within athletic populations (ideally elite) and their impact upon intermittent high-intensity, prolonged endurance and resistance based exercise (perceptual and performance orientated outcome measures), and cognitive function, within and next day.
- The efficacy of sleep hygiene and non-pharmacological sleep enhancing interventions (Halson, 2008, 2014; Fowler et al., 2015; Nédélec et al., 2015b) relative to the data generated in response to (ii) and (iii).

## Author contributions

All authors listed, have made substantial, direct and intellectual contribution to the work, and approved it for publication.

### Conflict of interest statement

The authors declare that the research was conducted in the absence of any commercial or financial relationships that could be construed as a potential conflict of interest.
